# New Biomarkers for Atherothrombosis in Antiphospholipid Syndrome: Genomics and Epigenetics Approaches

**DOI:** 10.3389/fimmu.2019.00764

**Published:** 2019-04-16

**Authors:** Chary Lopez-Pedrera, Nuria Barbarroja, Alejandra Mª Patiño-Trives, Eduardo Collantes, Mª Angeles Aguirre, Carlos Perez-Sanchez

**Affiliations:** ^1^Instituto Maimonides de Investigación Biomédica de Cordoba, Reina Sofia Hospital, Córdoba, Spain; ^2^Hospital Universitario Reina Sofía, Córdoba, Spain; ^3^Inflammatory and Systemic Autoimmune Diseases' Group, Instituto Maimonides de Investigacion Biomédica de Córdoba, Cordova, Spain; ^4^Department of Medicine, Universidad de Córdoba, Córdoba, Spain

**Keywords:** Antiphospholipid Syndrome, cardiovascular disease, genomics, microRNAs, therapy

## Abstract

Antiphospholipid Syndrome (APS) is an autoimmune disorder, characterized by pregnancy morbidity and/or a hyper coagulable state involving the venous or the arterial vasculature and associated with antiphospholipid antibodies (aPL), including anti-cardiolipin antibodies (aCL), anti-beta2-glycoprotein I (anti-ß2GPI), and Lupus anticoagulant (LA). In recent years there have been many advances in the understanding of the molecular basis of vascular involvement in APS. APS is of multifactorial origin and develops in genetically predisposed individuals. The susceptibility is determined by major histocompatibility complex (MHC). Different HLA-DR and HLA-DQ alleles have been reported in association with APS. Moreover, MHC II alleles may determine the autoantibody profile and, as such, the clinical phenotype of this disease. Besides, polymorphisms in genes related to the vascular system are considered relevant factors predisposing to clinical manifestations. Antiphospholipid antibodies (aPL) induce genomic and epigenetic alterations that support a pro- thrombotic state. Thus, a specific gene profile has been identified in monocytes from APS patients -related to aPL titres *in vivo* and promoted *in vitro* by aPL- explaining their cardiovascular involvement. Regarding epigenetic approaches, we previously recognized two miRNAs (miR-19b/miR-20a) as potential modulators of tissue factor, the main receptor involved in thrombosis development in APS. aPLs can further promote changes in the expression of miRNA biogenesis proteins in leukocytes of APS patients, which are translated into an altered miRNA profile and, consequently, in the altered expression of their protein targets related to thrombosis and atherosclerosis. MicroRNAs are further released into the circulation, acting as intercellular communicators. Accordingly, a specific signature of circulating miRNAs has been recently identified in APS patients as potential biomarkers of clinical features. Genomics and epigenetic biomarkers might also serve as indices for disease progression, clinical pharmacology, or safety, so that they might be used to individually predict disease outcome and guide therapeutic decisions. In that way, in the setting of a clinical trial, novel and specific microRNA–mRNA regulatory networks in APS, modified by effect of Ubiquinol treatment, have been identified. In this review, current and previous studies analyzing genomic/epigenetic changes related to the clinical profile of APS patients, and their modulation by effect of specific therapies, are discussed.

## Introduction

Antiphospholipid Syndrome (APS) is an autoimmune disorder, clinically characterized by pregnancy morbidity and/or a hypercoagulable state involving the venous or the arterial vasculature and associated with antiphospholipid antibodies (aPLs), including anti-cardiolipin antibodies (aCL), anti-beta2-glycoprotein I (anti-ß2GPI), and Lupus anticoagulant (LA).

Patients with APS have enlarged incidence of vascular damage, involving thrombosis, accelerated atherosclerosis, stroke or myocardial infarction, among others (1, 2). Numerous mechanisms have been postulated to contribute to the development of thrombosis in APS patients, comprising synergic effects of autoantibodies with pro-thrombotic molecules, adhesion receptors, inflammatory mediators, oxidative stress, netosis, and a plethora of intracellular signaling molecules.

Thus, a number of studies have established that aPLs provoke a pro-atherothrombotic status through the induced expression of both pro-thrombotic and pro-inflammatory molecules such as tissue factor (TF), vascular endothelial growth factor (VEGF) and its receptor Flt-1, as well as by inducing oxidative stress and mitochondrial dysfunction in monocytes and neutrophils, along with increased neutrophil extracellular traps (NETs) formation (3–5). Moreover, it has been demonstrated that endothelial cells (EC) express significantly higher amounts of adhesion molecules (ICAM-1, VCAM-1, and E-selectin) and other inflammatory mediators when incubated with aPL antibodies and ß2GPI *in vitro* (6, 7). Likewise, the incubation of ECs with antibodies reacting with ß2GP1 induce their activation, accompanied by the upregulation of TF, (8) adhesion molecules and IL-6, along with the alteration of the prostaglandin metabolism.

Genetic predisposition to APS and aPLs has been stated by different reports. Animal models and human studies have highlighted HLA associations with the disease and the occurrence of aPLs in APS patients. Specifically, different HL-DR and HLA-DQ alleles have been associated with APS. In addition, major histocompatibility complex (MHC) genes seems to influence not only autoantibody production but also disease expression itself (9).

Genetic polymorphisms have also been linked to thrombosis in APS patients, including variants of coagulation factors, anti-thrombotic and fibrinolytic molecules [i.e., FXIII, tissue factor pathway inhibitor (TFPI), type-I plasminogen activator inhibitor (PAI-1)] inflammatory mediators [i.e., tumor necrosis factor alpha (TNFα)], parameters related to platelet activity (i.e., platelet FC receptor IIa, platelet glycoproteins GP Ia/IIa and GP IIb/IIIa), endothelial factors (i.e., thrombomodulin), etc. (9). Besides, the Fcγ receptor as well as a β2-GPI-domain V polymorphism have been demonstrated to be relevant factors predisposing to APS (10, 11).

More recently, microarrays studies allowed the identification of APS and systemic lupus erythematosus (SLE) specific gene signatures explaining the pro-atherosclerotic, pro-thrombotic and inflammatory states in these autoimmune diseases (12). However, the modulation of gene expression has left significant gaps in our understanding of the development and progression of these co-morbidities in APS and SLE. Epigenetics, defined by the changes or modifications in DNA that influence phenotype without altering the genotype, present a new and entirely different mechanism of gene regulation. Several interrelated epigenetic and post-transcriptional regulatory mechanisms altered in numerous autoimmune and cardiovascular diseases are DNA methylation changes, histone modifications and microRNA activity, all of which act by altering gene and protein expression levels (13).

While extensive epigenomic studies have identified specific DNA methylation changes and histone modifications -linked to the development, the disease activity and even the organ involvement- in a closely related disease to primary APS, such as SLE, to date no studies have been developed to analyze those epigenetic alterations in APS patients and their contribution to cardiovascular disease.

Conversely, microRNAs, which markedly affect immune system and have an important role in the pathogenesis of numerous autoimmune and inflammatory conditions, have been demonstrated to act as main regulators of a number of gene targets involved in clinical features of APS, such as immune response, atherosclerosis and thrombosis (14).

This paper reviews genomic and epigenetic approaches (mainly focused on the role of microRNAs) used to deep into the mechanisms associated with vascular involvement in primary APS.

## Pro-thrombotic and Atherogenic Changes Induced by Antiphospholipid Antibodies on Immune and Vascular Cells

Subjects positive for LA, higher titers of anti-CL, and anti-ß2GPI antibodies (known as “triple positives”), have the highest risks for thrombosis (15). Moreover, various studies have demonstrated that triple-positive aPL patients usually have high titers of antibodies to the major ß2GPI epitope on domain I, which confer LA activity, associated with the highest risk for thrombosis (16).

Multiple molecules, essential in the hemostatic system, and acting as key players in thrombosis and atherosclerosis development, are altered in APS immune and vascular cells by effect of aPLs, including TF, the VEGF/Flt1 axis, several toll-like receptors (TLRs), annexins, protein disulfide isomerase, etc.

Thus, the expression of TF, the major initiator of the blood coagulation, was firstly described by our group to be significantly elevated in monocytes from APS patients with a previous history of thrombosis and related to the presence of high titres of aPLs (17). Thereafter, the molecular mechanisms underlying the aPL-induced increased expression of TF in monocytes were delineated, suggesting that aPLs induce TF expression in monocytes from APS patients by activating, simultaneously and independently, the phosphorylation of MEK-1/ERK proteins, and the p38 MAP kinase-dependent nuclear translocation and activation of NF-kappaB/Rel proteins (18). Parallel studies performed in ECs also concluded that p38MAPK phosphorylation and NFkB activation mediated the aPL-induced TF expression and function, along with the upregulation of IL-6, IL-8 and the inducible nitric oxide synthase (iNOS) (19).

Signaling TF activities are mainly mediated by the protease activator receptors (PARs), major mediators of thrombosis, hemostasis and inflammatory processes. Thus, it has been shown that TF complexes with coagulation factors VIIa or/and Xa induce PAR1 and PAR2 signaling. Some years ago, we provided evidence of increased expression of PAR-1 and PAR-2 in monocytes from APS patients with previous history of thrombosis (20). That study also demonstrated a correlation between PAR-2 levels and IgG aPL titers, as well as a parallel behavior of TF and PAR-2 expression, so that PAR-2 inhibition prevented the IgG aPL-induced TF expression. Overall, this study suggested that TF/PAR2 axis is directly involved in the pathogenesis of the thrombotic complications associated with APS.

Previous reports indicated a close relationship between TF and VEGF, protein involved in normal vascular development, as well as in pathologies related to inflammation and cardiovascular disease (21). Prior studies reported increased plasma levels of VEGF in APS patients (22). Later, it was shown that monocytes from APS patients expressed increased levels of both, VEGF and its receptor Flt1. Besides, these molecules were produced by monocytes when treated with aPLs, in a process involving the p38 MAPK signaling pathway (23). Thus, VEGF can be considered a regulatory factor in aPL-mediated monocyte activation and TF expression, contributing to the proinflammatory–prothrombotic status of APS.

Proteomic studies have demonstrated that aPLs are also responsible for the altered protein profile of APS monocytes, involving deregulated expression of annexin A1 (AnxA1), annexin A2 (AnxA2), ubiquitin-like protein Nedd8, Rho A protein, protein disulfide isomerase (PDI), and Heat shock protein-60 (Hsp60). These proteins were associated with a hypercoagulable state, as well as with autoimmune-related responses (24).

Toll-like receptors (TLR)-2 and−4 are membrane receptors known by their roles in the activation of immune and endothelial cells, pathogen recognition and production of cytokines. The TLR pathway is activated in APS patients, in which peripheral mononuclear cells show a significant increase in the gene expression of TLR2 and TLR4 that mediate aPL-induced vascular abnormalities (25). *In vivo* studies in mice and *in vitro* experiments in human monocytes and an endothelial cell line have shown that TLR2 and TLR4 mediate the inflammatory activation of monocytes and endothelial cells induced by aPLs (26, 27), suggesting that these receptors might be considered a therapeutic target to prevent the thrombotic effects of aPLs in APS.

aPLs can also activate platelets, inducing the expression of the fibrinogen receptor glycoprotein IIb/IIIa (GlIb/IIIa) as well as thromboxane B2 (TXB2), promoting its aggregation and thus contributing to the development of thrombosis (9).

Pierangely and coworkers further described other potentially significant antigenic targets for aPLs, which included prothrombin, tissue plasminogen activator (tPA), phosphatidylserine (PS), plasmin, annexin 2, activated protein (APC), thrombin, antithrombin III (AT-III), and annexin V (9). Finally, aPLs are associated to the development of atherosclerosis, so that *in vivo*, it has been demonstrated a direct correlation between serum levels of aCL and anti-ß2GPI antibodies and the occurrence of coronary syndrome, myocardial infarction and stroke (28). A recent study demonstrated a strong association among higher aPL-IgG titers and development of thrombotic events, as well as with the presence of early atherosclerosis, as demonstrated by increased intimae-media thickness (IMT) (5). *In vitro*, several studies confirmed the involvement of aPLs in the formation of the atheroma plaque, through the activation of endothelial cells and leukocytes, and the induction of foam cell generation, by facilitating the adsorption of oxidized low-density lipoproteins by monocytes (3).

## Role of Oxidative Stress in the Development of Atherothrombosis in APS

Various studies have proven that oxidative stress is involved in the pathophysiology of APS. Thus, an increased oxidative status has been demonstrated in plasma of APS patients, more significantly associated to the triple positivity for aPLs, so that those patients showed higher plasma levels of prostaglandin 2 and 8-isoprostane compared to healthy donors (29).

In a recent study (5) we showed an increased production of reactive oxygen species (ROS) by monocytes and neutrophils, accompanied by significant losses in mitochondrial membrane potential, which disturbed the redox status, and were related to both, the autoimmune condition and the inflammatory and pro-atherothrombotic status of APS patients. Accordingly, *in vitro* studies demonstrated that the binding of aPL-IgG to the monocytes provoked a redox- sensitive signaling pathway that controlled the prothrombotic phenotype. Other studies demonstrated that the generation of superoxide induced by aPLs in plasmacytoid dendritic cells and monocytes upregulate the expression of the TLRs −7 and −8 (30).

All that data underlies the relevant role of autoimmunity in the induction of an oxidative status in these patients, which further acts as an underlying mechanism promoting cardiovascular disease.

## Netosis in APS Patients: A New Mechanism of Thrombosis Stimulated by Antiphospholipid Antibodies

A number of studies have recognized that NETosis generation is associated with autoimmunity, deep vein thrombosis, tissue damage and atherosclerosis (31). In the setting of APS, Knight and co-workers have established a key role for neutrophil extracellular traps (NETs) in the development of thrombosis, so that they revealed, *in vivo*, high circulating levels of NETs in the plasma of APS patients, related to the occurrence of thrombotic events. Moreover, they demonstrated through *in vitro* studies that aPLs, especially those targeting ß2GPI, activate neutrophils to release NETs, thus predisposing to thrombosis (32, 33). These results open a new point of debate about the potential role of NETs as therapeutic targets.

## Genetic Risk Factors of Athero-thrombosis in APS

APS is strongly associated with genetic abnormalities (Table 1). Family and population studies have indicated that genetic factors play a key role in the etiopathogenesis of this disorder, suggesting the existence of a genetic predisposition to this disease, either when it presents as a primary disorder or within the context of SLE (34). This predisposition can be explained by the influence of genes at the major histocompatibility complex (MHC) locus and outside the MHC. Thus, certain human leukocyte antigen (HLA) alleles (HLA-DR and HLA-DQ) are strongly linked to the presence of aPL autoantibodies (35, 51, 52). The HLA allele most frequently associated with APS are HLA-DRB1^*^04 (DR4), DRB1^*^07 (DR7), DRB1^*^1302 (DR6), DRw53, DQA1^*^0102, DQA1^*^0201, DQA1^*^0301, DQB1^*^0302 (DQ8), and DQB1^*^0604/5/6/7/9 (34).

**Table 1:** Genetic risk factors associated with athero-thrombosis in APS.

**Gene**	**Genetic factor**	**Association with APS**	**References**
MHC	HLA-DR and HLA-DQ alleles	Presence of autoantibodies aPL	(32–35)
PAI-1	675 insertion/deletion 4G/5G	Occurrence of thrombosis	(36–38)
EPCR	T6333C	Lower prevalence in APS with arterial thrombosis	(39)
Prothrombin	G20210A	Not clear relationship with thrombotic events	(40–42)
TFPI	T33C C399T	Venous thrombosis	(43)
TNFa	G238A	Arterial thrombosis	(44)
GPIa GPIb-alpha	C807T Kozak TC polymorphism	Arterial thrombosis	(45) (46)
p-selectin	G1902A	Thrombotic events	(47)
PSGL-1	Tandem repeats (VNTR)	Thrombosis	(48)
B2-Glycoprotein	G796T G1004C	Production of autoantibodies	(49); (50)
TLR4	A896G C1196T	Lower prevalence in APS patients with thrombosis	(24)

*MHC, major histocompatibility complex; HLA, human leukocyte antigen; PAI-1, type 1 plasminogen activator inhibitor 1; EPCR, endothelial protein C receptor; TFPI, tissue factor pathway inhibitor; TNFα, tumor necrosis factor alpha; GPIa, glycoprotein Ia; PSGL-1, p-selectin glycoprotein ligand 1; TLR4, toll like receptor 4*.

Several of these HLA alleles determine the susceptibility to produce aPL (LA, aCL, and anti-β2GPI) antibodies to prothrombin, annexin V, phosphatidylethanolamine-phosphatidylserine, independently of the clinical context, primary APS or SLE. In fact, the same associations have been found among aPLs and the HLA system in primary APS and in APS secondary to SLE (35, 51, 52). In addition, the pattern of HLA associations is also influenced by the different ethnic groups (36, 53, 54).

Other genes, outside the MHC, contribute to the development of the disease. Thus, genetic variations of various components in the hemostatic system, favoring blood coagulation, may modulate the clinical manifestation of thrombosis (37):

1. A single nucleotide variation (SNV) of the type 1 plasminogen activator inhibitor (PAI-1) gen has been related to high levels of this inhibitor in plasma in APS patients. This is a common 4G/5G single nucleotide insertion/deletion variation in the promoter region 675 pb upstream from the start of transcription (675 insertion/deletion 4G/5G). Individuals with the 4G allele have higher plasma levels of PAI-1 than those with the 5G allele. High levels of PAI-1 have been found associated with high titers of aPLs in one third of the patients. In turn, 4G/5G genotype was associated with the occurrence of both types of thromboses, arterial and venous, especially with venous thromboembolism in patients with APS (38, 39, 55).
2. The protein C pathway is a key modulator of the blood coagulation, having an anticoagulant activity. In APS, there is evidence of acquired resistance to activated protein C (PC) induced by aPLs. Endothelial protein C receptor (EPCR) might have a procoagulant action by inhibiting the activation of PC. Elevated levels of soluble EPCR have been found in SLE, which suggests its role on the development of thrombotic manifestations. Several haplotypes of EPCR gene, PROCP, have been described according to the SNVs (H1-H4). Thus, T6333C, A6936G, G6147A, and all in common would correspond to H1, H3, H4 and H2, respectively. The PROCP T6333C and A6936G variations (H1 and H3) have been linked with EPCR levels and thrombosis. In addition, H1 haplotype has been associated with reduced sEPCR levels and increased levels of activated PC, which would have a protective effect against thrombosis (40). In this regard, a recent study showed a lower prevalence of the PROCR H1 haplotype in APS patients with arterial thrombosis (40).
3. The proteolytic cleavage of prothrombin leads to the generation of thrombin, the end- product of the coagulation cascade. The alterations involving prothrombin lead to multiple imbalances in hemostasis, since this factor has procoagulant, anticoagulant and antifibrinolytic activities. The prothrombin G20210A SNV is a common gene mutation where guanine is changed by adenine at position 20210, and it has been associated with an increased risk of venous thrombosis in the general population (41). The frequency of the G20210A mutation of prothrombin (F2) gene is has been observed significantly increased in APS patients with previous thromboses (42, 56). Thus, this mutation could be considered an important genetic risk factor for clinical manifestations in APS. However, other studies performed in aPL positive patients (SLE) did not find association among this SNV and the occurrence of thrombotic events (43).
4. Tissue factor pathway inhibitor (TFPI) (the natural inhibitor of TF) can regulate the blood coagulation through the inhibition of the tissue factor-activated factor VII complex. Decreased TFPI plasma levels have been related to deep vein thrombosis in APS (44). Several TFPI SNVs have been studied in APS patients with or without vein thromboembolism. Lincz et al., observed a significant relationship among both, the T33C and C399T variations (single nucleotide variations located in the intron region of TFPI gene) and venous thrombosis in APS patients (45). Therefore, TFPI SNV is another genetic risk factor for the development of thrombosis in APS.
5. Tumor necrosis factor alpha (TNFα) is a cytokine with a well-recognized role in inflammation. Bertolaccini et al., reported high levels of this proinflammatory molecule in patients with APS. In addition, they showed an increased frequency of the G238A SNV of TNFα gene, strongly related to arterial thrombosis, suggesting the importance of this genetic marker for the development of thrombosis (46).
6. Glycoprotein (GP) Ib and GP Ia/IIa bind to von Willebrand factor (vWF) and collagen, respectively, to mediate the adhesion of platelets to the vascular wall. The presence of SNVs in these genes might increase the platelet adhesion and aggregation, predisposing to the development of arterial and/or venous thrombosis. Thus, the frequency of platelet GPIa C807T SNV has been shown higher in APS patients having thrombosis compared to those without thrombosis or controls. In addition, the frequency of the T/C polymorphism in the kozak sequence of GPIb-alpha has been found increased in APS patients with arterial thrombosis compared to APS patients with venous thrombosis, or APS patients without thrombosis. That data suggest that these two polymorphisms may be responsible for the thrombosis occurrence in APS patients (47, 57).
7. P-Selectin is a cell adhesion molecule that mediates the attachment and rolling of leukocytes on activated endothelial cells and the recruitment of leukocytes to the thrombi. Plasma levels of p-selectin are increased in APS patients (48). A study of 40 APS patients showed an increased prevalence of a single nucleotide variation associated with the coding region of p-selectin, G1902A, compared to healthy controls. Among APS patients, those who had thrombotic events presented more significantly augmented prevalence of this genotype compared to APS without thrombosis (49). Moreover, the interaction of p-selectin on activated platelets or endothelial cells with the p-selectin glycoprotein ligand (PSGL-1) on monocytes is considered relevant in processes such as inflammation and thrombosis, since it can activate TF expression. PSGL-1 presents several tandem repeats (VNTR) polymorphisms. This genotype frequency is elevated in APS patients, especially in those with thrombosis (50). These studies pointed out to selectin-PSGL1 system genetic variations as determinant of thrombotic predisposition in patients with APS.
8. Two single nucleotide variations in β2-Glycoprotein gene have been found increased in APS: G796T and G1004C, more known by the changes in aminoacidic sequence; Val247Leu and Trp316Ser, respectively (58, 59). The presence of these SNVs may lead to a conformational change in β2GPI that affects the exposure of potential epitopes, which in turn may favor thrombosis. Previous studies suggested that the presence of Val247Leu genotype is related to the production of anti- β2GPI antibodies. However, the relationship with either arterial or venous thrombosis is not clear (59). In the same way, the presence of Trp316Ser SNV may increase the risk of APS, but there is no relationship with thrombosis or antibodies production (58). Thus, further studies might be required to evaluate the pathogenic link between these SNVs and thrombosis in APS.
9. Pierangeli et al. evaluated the prevalence of two SNVs in Toll-like receptor 4 (TLR4) gene in 100 APS patients: A896G and C1196T. Both of the TLR4 SNVs confer an alteration to the extracellular domain of the TLR4 receptor, which may affect the binding of ligands. The frequency of these two SNVs was significantly reduced in APS patients with thrombosis compared to healthy donors (26). These two TLR4 SNVs are supposed to be protective against thrombosis. Thus, decreased prevalence of these polymorphisms in APS patients might suggest a higher susceptibility to an aPL-mediated procoagulant endothelium activation (26).

Considering all these genetic alterations associated with APS (at the MHC locus and outside the MHC), it is becoming increasingly clear that interactions between more than one genetic abnormality could determine whether an individual will develop the disease or suffer from the different clinical manifestations.

## Gene Expression Signatures Associated to CVD in APS

As detailed in the first part of this review, in the last years a number of studies have identified several genes involved in thrombosis, inflammation and endothelial dysfunction altered in APS patients (i.e., TF, PAR1, PAR2, VEGF, Flt1, IL8, TLR2, TLR4, etc.), most of them showing increased expression in cells integrating the immune and vascular systems, including monocytes, platelets, neutrophils and endothelial cells, favoring the thrombin generation, and leading to a procoagulant activity (17, 20, 23, 25–27).

Patsouras et al. (60) further reported a significant increase of gene expression of the platelet factor (CXCL4) and its variant, CXCL4L1, in platelets of APS patients compared to the control group and to SLE patients. CXCL4 and CXCL4L1 are chemokines produced by the platelets during the aggregation. They are involved in a variety of biological processes, including inflammation, blood coagulation, and angiogenesis. They have also pro-coagulant effects and anti-angiogenesis activity, inducing angiostasis by inhibiting endothelial cell proliferation and chemotaxis. That study showed that APS patients having high levels of CXCL4/CXCL4L1 in plasma were characterized more often by IgG aCL, double antibody or triple antibody positivity, and presented more than 3 thrombotic events, thus underlying the relevance of the increased expression of these molecules in the occurrence of thrombosis.

Recent advances in gene expression analysis allowed to perform broad-based gene expression profiling. Thus, a study by Hamid C and coworkers (61), using Affymetrix Human Genome U133A-2.0 arrays, revealed a complex gene expression response in HUVECs to *in vitro* treatment to anti- β2GPI antibodies, involving multiple chemokines, pro-inflammatory cytokines, pro-thrombotic and pro-adhesive genes. Moreover, that study showed that some of these newly identified anti-β2GPI antibody-regulated genes could contribute to the vasculopathy associated with this disease.

Recently, Ripoll VM and colleagues analyzed the *in vitro* effects of aPLs from thrombotic or obstetric APS patients in monocytes, in order to identify different molecular pathways related to the pathogenesis of the APS subtypes (62). Thus, genes related to cell response to stress, MAPK signaling modulation and cell interactions were induced by IgGs isolated from patients with vascular thrombosis. In contrast, genes associated with cell adhesion, extracellular matrix and embryonic and skeletal development were modulated by IgGs purified from patients with pregnancy morbidity, suggesting that the IgGs from the different clinical subtypes of APS induce disease-specific genome profiles in monocytes, associated with different physiological mechanisms.

Using microarray technology, our group identified, *in vivo*, shared and differential genetic patterns related to atherosclerosis and cardiovascular disease in APS, APS plus SLE, and SLE patients. Thus, the gene expression analysis of monocytes led to the segregation of APS, APS plus SLE and SLE, with specific profiles associated with the pro-atherosclerotic, pro-thrombotic and inflammatory alterations. The specific features of APS monocytes comprised genes involved in mitochondria biogenesis and function, oxidative stress and antioxidant defense, processes directly related to the development of thrombosis in APS (12). In addition, those alterations were related to the levels of aPLs, a fact that was demonstrated with *in vitro* studies, where treatment of healthy monocytes with aPLs modulated the expression of genes involved in such processes including CCL2, IFIT1, PPAR gamma, SLC25A27, ARHGEF5, and IL11RA (12).

Finally, a very recent systematic review, by using bioinformatic analyses, identified a number of genetic risk factors in thrombotic APS (37). They found that sixteen genes (CXCL4L1, P-Selectin, TLR2, TLR4, PAI-1, β2GPI, GP1a, GP1BA, PAR1, PAR2, TFPI, TF, VEGFA, FLT1, TNF, and Prothrombin) contribute significantly, while six (PLSCR1, PTPN22, ACAPMTS13, F13A1, ACE, and F5) were not associated with thrombosis in primary APS. These genes affected mostly the immune system and blood coagulation pathways. Moreover, the authors suggested that these genes, expressed in 32 different organs, may pose higher risk of developing thrombosis anywhere in the body of primary APS patients.

**Figure 1:**
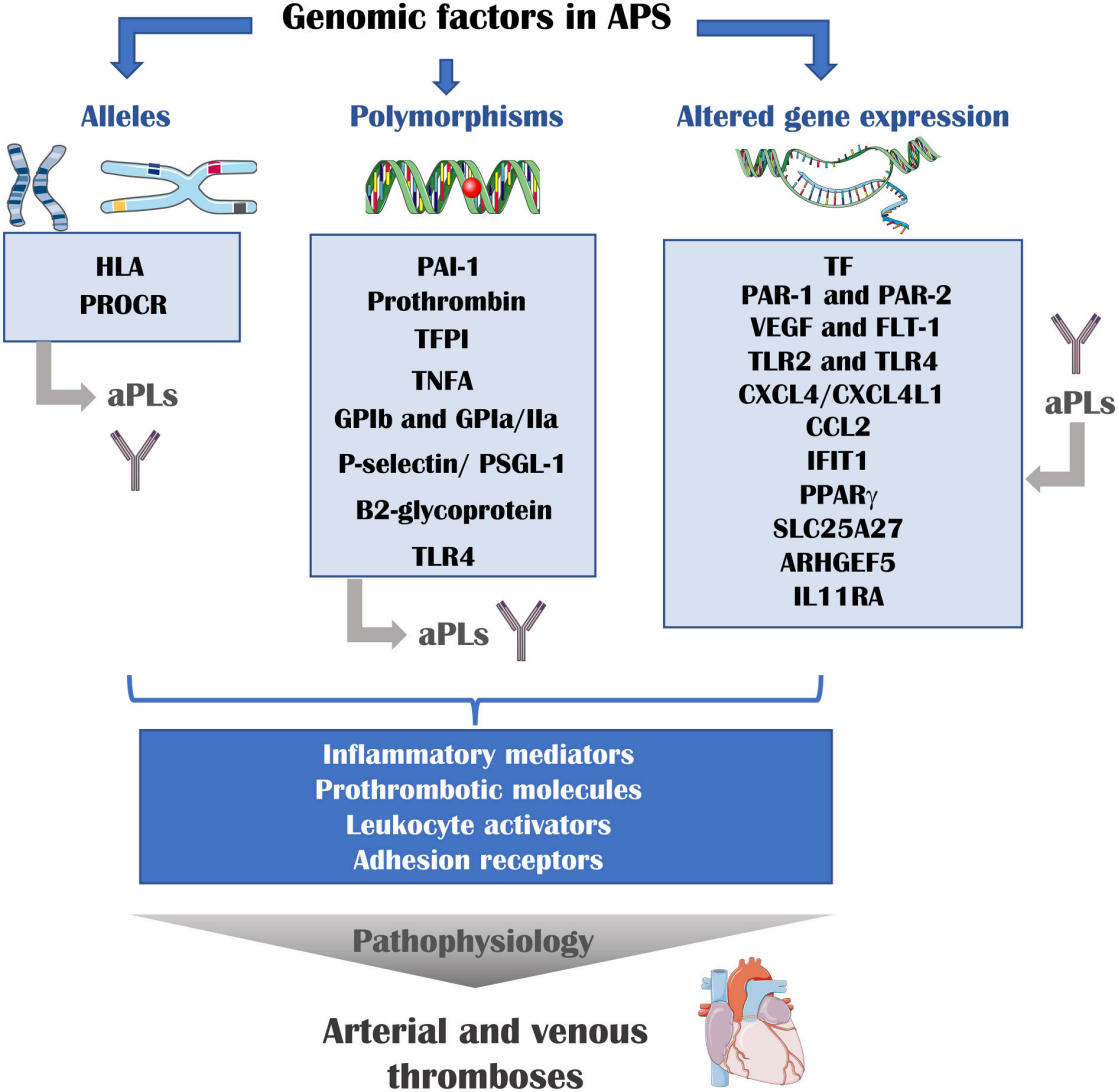
Genomic risk factors of athero-thrombosis in APS. Multiple genomic factors are involved in the pathophysiology of APS. The presence of determined alleles or polymorphisms is associated with the presence of aPLs and thrombotic events. In addition, aPLs can modulate the expression of several genes. These genes encode for inflammatory mediators, prothrombotic molecules, leukocyte activator and adhesion receptors, proteins that are directly involved in the development of thrombosis. HLA, human leukocyte antigen; PROCR, endothelial protein C receptor gene; PAI-1, plasminogen activator inhibitor 1; TFPI, tissue factor pathway inhibitor; TNFA, tumor necrosis factor A; GP, glycoprotein; TLR, toll-like receptor; TF, tissue factor; PAR, protease activator receptor; VEGF, vascular endothelial growth factor; Flt-1, VEGF receptor 1; CXCL4, platelet factor 4; CXCL4L1, platelet factor variant 1; CCL2, C-C motif chemokine ligand 2; IFIT1, interferon-induced protein with tetratricopeptide repeats 1; PPAR gamma, peroxisome proliferator-activated receptor gamma; SLC25A27, Solute Carrier Family 25 Member 27; ARHGEF5, Rho guanine nucleotide exchange factor 5; IL11RA, interleukin 11 receptor subunit alpha.

Overall data suggest that a complex network of genetic factors (involving altered gene expression mainly induced by aPL, multiple alleles and polymorphisms) including inflammatory mediators, oxidative stress, prothrombotic molecules, leukocyte activators, and adhesion receptors are responsible for the APS pathophysiology (Figure 1).

## Epigenetic Mechanisms Underlying the Pathophysiology of APS and the Development of Atherothrombosis

Since genome-wide profiling does not give a sufficient resolution to explain the complex pathological features of patients with autoimmune disorders, epigenetic modifications are engaged additional regulators in immune responses. Epigenetic mechanisms, known for their ability to regulate gene transcription and genomic stability, are key players for maintaining normal cell growth, development, and differentiation (13). The term “epigenetics” can be defined as the heritable alterations in gene expression, related to environmental factors, without changes in the sequence of bases in the DNA.

Epigenetic modifications can be broadly categorized into: (1) Histone modifications -including methylation, acetylation, phosphorylation ubiquitination, ADP ribosylation, and sumoylation- (2) DNA methylation and emerging RNA methylation; and (3) Non-coding RNAs mechanisms, such as microRNAs (63). Distinct from genetic mutations, epigenetic alternations are reversible, and vulnerable to nutritional and environmental factors, and thus more manageable for modification and/or drug targeting (64).

In the last years, several new findings about epigenetic modifications of gene expression have been reported in different autoimmune disorders. These modifications designate changes in the expression of DNA that result from methylation, posttranslational modifications of histone proteins, i.e., acetylation/deacetylation, methylation, and microRNAs. Remarkably, these modifications seem to act jointly (65).

### Histone Modifications

Histone modification refers to the post-transcriptional modification of a specific amino acid in the polypeptide side-chain of a histone protruding from the nucleosome. Histone modification affects local chromatin conformation altering its accessibility, and thus influencing gene transcription activity. Histone modifications are coordinated in cellular processes such as the cell cycle, development, and differentiation (66).

They include acetylation, methylation, ubiquitination, and sumoylation, each with different function and biological significance. To date, histone acetylation and histone methylation are the best studied processes (67).

Acetylation of histones induces the relaxing of compacted chromatin and grants the access of transcription factors to gene promoter regions, while deacetylation of terminal lysine residues silences the transcription. In this process, a number of identified histone acetyl transferases (HAT, including PCAF, Tip60 and p300/CBP) add the acetyl group, and the histone deacetylases (HDAC, including HDAC1 and sirtuins) remove it. Thus, HATs and HDACs coordinately regulate the acetylation status of proteins, so that changes in HAT/HDAC activity influence the cellular gene transcription in response to extracellular stimuli.

Likewise, the methylation of lysine or arginine residues by histone methyltransferases (HMTs) is reversed by demethylating enzymes (i.e., lysine-specific demethylase 1 and JmjC domain-containing histone demethylase). This reversible property of histone modifications provides great potential for the modulation of these epigenetic mechanisms (68).

SLE is the paradigm of disease on which epigenetic abnormalities and their patterns of inheritance are exceptionally complex. Global H3 and H4 hypoacetylation and hypermethylation characterize CD4+ T cells from active SLE patients compared with CD4+ T cells from patients with inactive SLE and healthy individuals, indicating that histone H3 and H4 acetylation inversely correlates with disease severity (69). Furthermore, there are a number of clusters comprising aberrantly expressed genes in SLE (including those codifying for a set of chemokines) strongly associated with altered H4 acetylation (70).

To date, no studies have been developed to analyze histone modifications in primary APS patients.

### DNA Methylation Alterations

DNA methylation is a relatively stable and heritable epigenetic mark present in eukaryotic organisms. It refers to the addition of a methyl group to the 50 carbon in the pyrimidine ring of a cytosine residue, usually occurring in the context of cytosine-guanine dinucleotides (CpG) in mammal DNA. Although CpG dinucleotides are present throughout the whole DNA sequences and represent only approximately 4% of the human genome, research interest in DNA methylation has been mainly focused on those CpG sites located within the 50 upstream promoter regions of genes. The methylated status of CpG sites in a promoter region blocks the accessibility to transcriptional activators and thus inhibits the gene transcription, serving as a repressive “lock,” while an unmethylated state at the promoter permits transcription.

The methylation process is catalytically mediated by DNA methyltransferases (DNMTs), which mainly include DNMT1, DNMT3a, and DNMT3b. DNMT1 acts as a maintenance methyltransferase enzyme that recognizes and copies the preexisting methylation profiles of a DNA strand to the new strand during DNA replication in the S phase of the cell cycle, whereas DNMT3a and DNMT3b induce *de novo* methylation (71).

Differential DNA methylation is a known epigenetic feature of SLE that not only differentiates SLE from healthy individuals, but also correlates with various organ-specific manifestations, and may play a dynamic role in disease activity via mediation of T helper cell response, among other pathophysiologic mechanisms (72). Candidate gene studies have identified several pathways in which aberrant gene expression promoted by DNA demethylation is closely related to the development of SLE (73–76). Epigenomics has further suggested that specific DNA methylation changes in lupus CD4+ T cells are correlated with different clinical phenotypes of SLE such as skin lesions only, skin and renal involvement only, and skin and renal involvement with polyarticular disease, while there are also common methylation changes found in all groups of SLE compared to controls (77).

One of most relevant advances in this field has been the identification of IFN-induced protein 44-like (IFI44L) promoter methylation in peripheral blood cells as a biomarker with high sensitivity and specificity for diagnosis of SLE superior to that of other available tests (78). Besides, the IFI44L promoter methylation level may also be a potential biomarker of disease activity of SLE, because this gene promoter exhibit significantly increased methylation level in lupus patients in remission stage than in active stage. Moreover, the IFI44L promoter methylation also shows significantly lower level in SLE patients with renal damage than those without renal damage (78).

Likewise, genome-wide studies of DNA methylation have identified many differential methylation loci in T and B lymphocytes from SLE patients compared to those from healthy individuals, with methylation status of several genes correlating with disease activity (i.e., RAB22A, STX1B2, LGALS3BP, DNASE1L1, and PREX1), (79).

Previous studies further demonstrated epigenetic aberrancies in SLE neutrophils, with demethylation in a number of interferon-regulated genes (80). Thereafter, a proinflammatory transcriptional signature in APS neutrophils was demonstrated, suggesting a role of neutrophils in the pathogenesis of APS (81).

Although the DNA methylation profile of APS remains to be characterized, a very recent study examined the genome-wide DNA methylation signatures in neutrophils from individual with primary APS vs. healthy individuals and compared the differential methylation profile of primary APS to that of SLE (82).

Seventeen hypomethylated and 25 hypermethylated CpG sites were identified in relation to healthy donors. Remarkable hypomethylated genes included ETS1, a genetic risk locus for SLE, and PTPN2, a genetic risk locus for other autoimmune diseases. Gene ontology analysis of the hypomethylated genes revealed enrichment of genes involved in pregnancy. None of the differentially methylated sites in primary APS were differentially methylated in SLE neutrophils, and there was no demethylation of interferon signature genes in primary APS as is seen in SLE. Furthermore, no other differentially methylated genes or gene regions were shared between the two disease methylation profiles.

The most notable finding of this study was the association between differential methylation in APS and genetic regions regulating pregnancy, a defining feature of APS. Thus, gene ontology analysis revealed enrichment of hypomethylated genes known to be associated with human pregnancy, namely ETS1, EMP2, and OXT. Hypomethylation of ETS1 and EMP2 in primary APS neutrophils may indicate dysregulation of trophoblast differentiation and migration which could contribute to increased fetal morbidity. DPPA3, also hypomethylated in primary APS, plays an important role in embryogenesis in murine and bovine models, though its role in humans is less clear. Functional studies of how DNA methylation affects these genes and their associated cellular functions may elucidate the mechanisms by which APS causes pregnancy morbidity and potentially guide future treatment strategies.

As in the case of histone modifications, the DNA methylation profile of APS remains to be characterized in leukocyte subsets of APS patients, as well as their involvement in the development of atherothrombosis.

### Cellular miRNAs as Biomarkers of Disease in APS

Current genome-wide studies have shown that the human genome is extensively transcribed and produces many thousands of regulatory non-protein-coding RNAs (ncRNAs), including miRNAs, small interfering RNAs, and various classes of long ncRNAs. It is now clear that these RNAs fulfill critical roles as transcriptional and post-transcriptional regulators and as guides of chromatin-modifying complexes. Among them, miRNAs are small ncRNAs ubiquitously expressed, with a profound influence in the regulation of almost every cellular process investigated, and whose expression changes are observed in numerous human pathologies (83).

MicroRNAs (miRNAs) control posttranscriptional expression of genes by destabilizing target transcripts or by inhibiting protein translation (84). In humans, more than 2,500 miRNAs been described so far, and the number is still increasing. They act as potential modulators of the transcription of more than 20,000 genes encoding human proteins. The first step in the canonical pathway of miRNA biogenesis is the transcription of miRNA genes by RNA polymerase II/III. The pri-miRNA is cleaved by the microprocessor complex Drosha and DGCR8 to generate the pre-miRNA. Exportin 5 protein facilitates the exportation of the pre- miRNA to the cytoplasm, where it is again processed by the RNase Dicer. This enzyme produces a miRNA duplex of 5 p and 3 p strands of 22 base pairs approximately. Finally, one strand is loaded into the proteins Argonaute (AGO), generating the complex miRISC (miRNA-induced silencing complex). In recent years, non-canonical pathways for miRNA biogenesis are further emerging, including those that are independent of Drosha or Dicer (85, 86).

miRNAs have been demonstrated to control a wide range of physiological functions such as embryogenesis, cellular differentiation, proliferation, cytokine production and apoptosis. Furthermore, their altered levels have been associated to a number of pathophysiological processes such as cancer, cardiovascular disease, viral infections, neurodegenerative diseases and immune-related diseases, among others (87).

The first study that intended to characterize the role of miRNAs in the pathogenesis of APS was published in 2011 (88) (Table 2). That study identified two miRNAs as main regulators of the expression of TF, as mentioned above, a key procoagulant molecule involved in the development of thrombotic complications in APS. Thus, the expression levels of miR-19b and miR-20a by RT- PCR in monocytes from APS and SLE patients were found significantly reduced when compared with healthy donors and negatively correlated with the increased expression of TF in the cell surface of monocytes from both APS and SLE patients. This study suggested for the first time the potential role of those miRNAs in the pathogenesis of thrombosis in APS patients. However, the mechanism by which both, miR-19b and miR-20a are reduced in monocytes from APS and SLE patients remains to be clarified.

**Table 2:** microRNAs differentially expressed in Antiphospholipid Syndrome patients.

**microRNA**	**Alteration**	**Source**	**Technique**	**References**
miR-19b, miR-20a	Reduced	Monocytes	RT-PCR	(88)
miR-124a-3p, miR-125a-5p,miR125b-5p, miR-146a-5p,miR-155-5p, miR-222-3p	Reduced	Neutrophils	RT-PCR	(89)
miR-124a-3p, miR-125a-5p	Reduced	Monocytes	RT-PCR	
miR-155-5p, miR-146a-5p	Increased	Monocytes	RT-PCR	
miR-146a-3p	Increased	Exosomes (plasma)	RT-PCR	(90)
miR-299-3p, miR-579,miR-494, miR-221-3p,miR-4516, miR-145-5p,miR-146b-5p, miR-371a-3p,miR-18a-5p, miR-26a-5p,miR-199a-5p, miR-376c,miR-126-3p, miR-7f-5p,miR-30b-5p, miR-106a-5p	Reduced	Monocytes	Nanostring	(91)
miR-29a-3p, miR-451amiR-150-5p	Increased	Monocytes	Nanostring	
miR-19b/miR-34a,	Increased	Plasma	RT-PCR	(92)
miR-19b/miR-15a, miR-19b/miR-124, miR-19b/miR-145, miR-20a/miR-145, miR-20a/miR-374a, miR-20a/miR-210, miR-20a/miR-133b, miR-206/miR-34a				
miR-124/miR-296	Reduced	Plasma	PCR-Array	
miR-125b, miR-99a, miR-99b, miR-127, miR-181a, miR-590-3p, miR-744,miR-27a, miR-30a-5p, miR-126,miR-30e-3p, miR-335, miR-27b,miR-20a, miR-29a, miR-942,let-7c, let-7f, let-7g, let-7e, let-7a	Reduced	Plasmacytoid dendritic cells	Open-Array	(93)

In a later study, we identified and characterized a number of miRNAs related to the cardiovascular disease present in APS and SLE patients (89) recognized as the main regulators of targets involved in clinical features of APS such as atherosclerosis, thrombosis, immune response and oxidative stress: miR-124a-3p, miR-125a-5p, miR-125b-5p, miR-146a-5p, miR-155- 5p, and miR-222-3p. The levels of these miRNAs were reduced in neutrophils purified from both, APS and SLE patients in relation to the control group. Moreover, the expression of molecules related to the miRNA biogenesis such as Dicer, Drosha, Exportin-5, Argonaute-1 and Argonaute-2, were decreased in both groups of patients. In monocytes isolated from APS and SLE patients, a reduction in the levels of miR-124a and miR-125a was found, while miR-155 and miR-146a appeared increased. Furthermore, the altered levels of both, miRNAs and their biogenesis proteins, correlated with markers of thrombosis, inflammation and oxidative stress, and were associated to the presence of thrombotic events, as well as with an increased Carotid Intimae Media Thickness (CIMT). The *in vitro* treatment of monocyte and neutrophils, isolated from healthy donors, with aPL-IgG purified from the serum of APS patients promoted an alteration of the levels of selected miRNAs, along with those of their biogenesis proteins. The pathogenic role of aPLs in miRNA expression was also supported by the fact that SLE patients positive for aPLs displayed a specific dysregulation of miRNAs in relation to those without such autoantibodies. In addition, a parallel cohort of 20 non-autoimmune (aPL negative) patients with previous thrombotic events showed differential miRNAs alteration than thrombotic APS patients (aPL positive). Taken together, these results established that: (1) Specific miRNAs might be considered potential biomarkers of immune activation and atherothrombotic development in APS. (2) Antiphospholipid antibodies are involved in the deregulated expression of both, miRNAs related to cardiovascular disease, and biogenesis proteins in leukocytes from APS and SLE patients.

**Figure 2:**
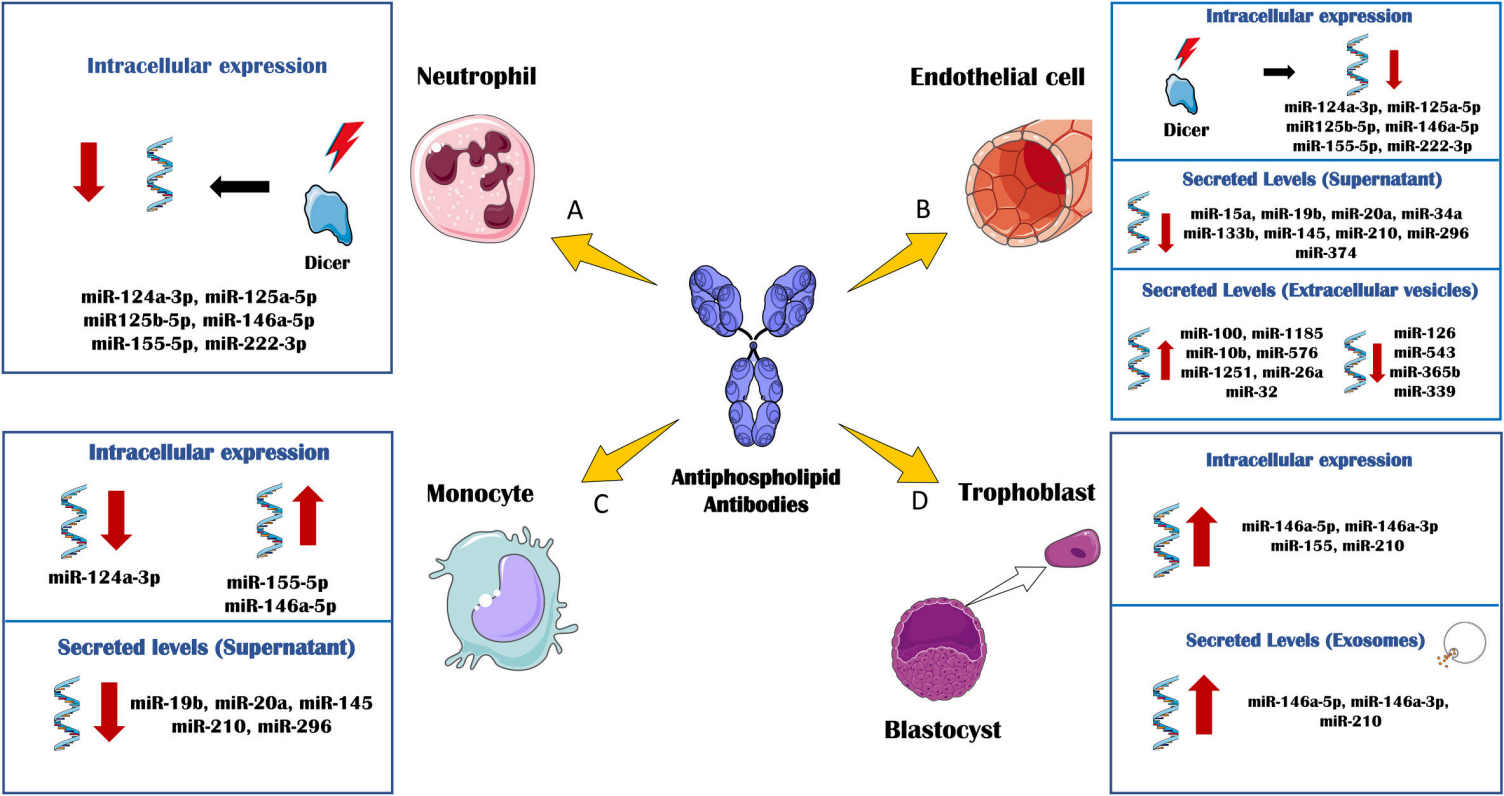
*In vitro* effects of antiphospholipid antibodies (aPL) through miRNA biology. The *in vitro* treatment of several immune and vascular cells with aPL, has allowed delineating the regulation of cellular and extracellular levels of miRNAs associated to the APS pathology. **(A)** aPL- IgG treatment of neutrophils purified from healthy donors promoted the down-regulation of DICER and other related miRNA biogenesis proteins. Accordingly, the intracellular levels of several miRNAs, including miR-124a-3p, miR-125a-5p, miR125b-5p, miR-146a-5p, miR-155-5p, and miR-222-3p were also reduced. **(B)** Human umbilical vein endothelial cells (HUVEC) cultured in the presence of aPL-IgGs, showed a downregulation of DICER and miRNA biogenesis proteins along with the intracellular levels of miR-124a-3p, miR-125a-5p, miR125b-5p, miR-146a-5p, miR- 155-5p, and miR-222-3p. aPLs also induced effect through the circulating miRNA profile. The secreted levels of miR-15a, miR-19b, miR-20a, miR-34a, miR-133b, miR-145, miR-210, miR-296, and miR-374 were found reduced in the supernatant of HUVECs treated with aPL-IgGs compared to IgG control. The treatment of HUVECs with ß2GPI antibodies promoted the secretion of extracellular vesicles whose miRNA profile was increased in miR-100, miR-1185, miR-10b, miR- 576, miR-1251, miR-26a and miR-32, and reduced in miR-126, miR-543, miR-365b and miR-339, in relation to the IgG-control treatment. **(C)** aPL-IgGs treatment on monocytes isolated from healthy donors, promoted the reduction of the intracellular levels of miR-124a-3p while the levels of miR-155-5p and miR-146a-5p were up-regulated. The secreted levels of miR-19b, miR- 20a, miR-145, miR-210 and miR-296 were reduced in the supernatant of healthy monocytes treated with aPL-IgGs purified from APS patients compared to the control-IgGs treatment. **(D)** The culture of human first trimester trophoblast cell line, HTR8, in the presence of b2-GPI antibodies elevated the intracellular levels of miR-146a-5p, miR-146a-3p, miR-155 and miR-210. In parallel, secreted exosomes derived of the ß2GPI antibodies treatment on these cells, showed augmented levels of miR-146a-5p, miR-146a-3p, miR-210.

In a very recent study (93), van den Hoogen L et al. analyzed the expression profiles of miRNAs and mRNAs in plasmacytoid dendritic cells (pDCs), the major producers of IFN-gamma in APS and SLE. A global reduced expression of miRNAs in all groups of patients was found, further related to their activation status, whereas the miRNA profiles among patients with SLE, SLE + APS and primary APS did not show strong differences. The global miRNA downregulation seemed not to be due to alterations in the miRNA machinery, since the miRNA biogenesis proteins did not show altered expression. However, pDC miRNA expression was related to the type I IFN signature in pDCs, so that miRNA expression was strongly reduced in patients with an IFN-high signature. Three miRNAs (miR-361-5p, miR128-3p, and miR-181-2-3p) were expressed at lower levels in IFN-high patients and found downregulated in pDCs activated by TLR7 agonist R837 (imiquimod). Pathway enrichment analysis revealed that the genes upregulated and predicted as target of these three miRNAs were involved in pDC activation and apoptosis. These finding suggested that aberrances in miRNA expression may have a key role in regulating pDC activity and the immunopathology of SLE and APS (Figure 2).

### Circulating miRNAs as Biomarkers of Disease in APS

Publications involving circulating miRNAs as diagnostic and prognostic biomarkers in many diseases have grown exponentially over the past decade. miRNAs are present in almost all human body fluids (including blood, plasma, serum, saliva, urine, seminal fluid, and pleural effusion) as a consequence of either, necrotic or apoptotic cell death, or an active release. Growing evidence highlights the role of miRNAs in cell-to-cell communication, having the capacity of regulating gene expression outside of the cell of origin. Circulating miRNAs are encapsulated in exosomes and/or bound to proteins and lipoproteins, and thus protected from endogenous RNAses, which make them stable and suitable for non-invasive analysis in patient samples (94). It has been shown that the circulating profile of miRNAs might have potential as biomarker of diagnosis, therapeutic response and prognosis in a wide range of cardiovascular pathologies and autoimmune diseases such as systemic sclerosis, rheumatoid arthritis, and SLE. With this in mind, we recently analyzed the circulating miRNA signature of APS and their potential role as biomarkers of disease and atherothrombotic status in a cohort of 90 patients (92) (Table 1). MicroRNA expression profiling identified 39 miRNAs differentially expressed in APS, including 19 increased and 20 reduced. Bioinformatic tools allowed to identify a set of them that showed potential mRNA targets involved in the physiopathology of APS. Eleven miRNAs were validated in the whole cohort of patients, including miRNAs 34a-5p, 15a-5p, 133b-3p, 145a-5p, 124-3p, 20a-5p, 19b-3p, 210-3p, 206, 296-5p, and 374a-5p. The signature generated by these miRNAs allowed to identify APS patients with a marked accuracy (AUC of 0.81), was found associated to the presence of both, fetal loss and type of thrombosis, and correlated with parameters related to inflammation and thrombosis (TF, PAI-1, VEGF-A, VEGF-R1, and MCP-1). In line with these findings, hard clustering analysis differentiated 3 clusters of APS patients representing different thrombotic risk groups, and significant differences between groups for several miRNA ratios were found. Among them, the ratios generated by the miR-124, miR-19b, and miR-296 were associated with an increased CIMT, thus recognizing APS patients with early atherosclerosis. These results showed the potential role of circulating miRNAs as biomarkers of atherothrombotic in APS.

We could also prove that the plasma miRNA signature remained stable over time after the analysis of samples from the same patients 3 months after the first sample collection. In addition, the specificity of the miRNA signature in APS was also confirmed in relation to both, SLE-aPL negative patients and thrombotic non-autoimmune patients.

A significant correlation between the circulating miRNA signature in APS and the titers of aPL was also noticed. Moreover, *in vitro* treatment of healthy monocytes and EC with IgG-aPL purified from the serum of APS patients, promoted a deregulated secretion of miRNAs and target proteins into the culture supernatant. These results reinforced the pathological effects of these autoantibodies, which also modulate the circulating miRNA profile found in APS patients related to their atherothrombotic status.

Similarly, a study conducted by Wu et al. (95), revealed that the treatment of ECs with anti β2GPI antibodies isolated from APS patients promoted the secretion of extracellular vesicles (EVs), whose miRNA content was different from that secreted after treatment with a non- immune-IgG. The treatment with anti-β2GPI antibodies induced the expression of miR-100, miR-1185-1, miR-10b, miR-576, miR-1251, miR-26a and miR-32, while the levels of miR-126, miR-543, miR-365b, and miR-339 were found down-regulated. The EVs secreted after aPLs exposition, were enriched in IL1β and inflammasome components which, in turn, were able to activate unstimulated endothelial cells. Thus, that study suggested that alterations in miRNA profile may contribute to the ability of EVs, derived from endothelial cells treated with aPLs, to activate unstimulated endothelial cells in an autocrine or paracrine manner.

Interestingly, circulating miRNAs might also have a potential role as biomarkers in obstetric APS. Gysler et al. (90) identified that patients with aPLs and adverse pregnancy outcomes expressed significantly higher levels of circulating miR-146a-3p compared with “healthy pregnant.” Moreover, the *in vitro* treatment of a trophoblast cell line with aPLs from APS patients significantly increased cellular and exosome expression of miRs associated to TLR signaling, including miR-146a-5p, miR-146a-3p, miR-155, and miR-210. It was also established that the upregulation of miR-146a-3p contributed to the pathogenesis of obstetric APS, driving the cells to secrete interleukin (IL)-8 by activating the RNA sensor, TLR8 (Figure 2).

### Specific miRNAs Display Common Roles in Autoimmune and Cardiovascular Diseases

Several miRNAs displaying an altered expression in APS have been previously shown to play critical roles in the development of other autoimmune and inflammatory disorders, as well as in the pathogenesis of several cardiovascular diseases. Thus, miR-146 and miR-155 are key modulators of both innate and adaptive immune responses. Both miRNAs modulate the expression of several TLR4 effectors, such as IRAK1, IRAK2, TRAF6, IRF3, IRF5 (miR-146a), SHIP1, SOCS1, TNFα, and PU.1 (miR-155). Deregulated miR-146a and miR-155 expression have been associated with several chronic inflammatory disorders, such as SLE, RA, periodontitis, nephropathy and atherosclerosis (96). Other miRNAs such as miR-124a and miR-125 have been also previously reported as modulators of targets involved in the inflammatory chemokine pathway such as MCP1 (miR-124) or RANTES (miR-125). Furthermore, their expression was also found altered in other systemic autoimmune diseases, including SLE and RA (97, 98). The miRNA cluster 17–92, which includes miR-19 and miR-20, has been linked to different cardiovascular pathologies. Through the modulation of key proteins like MAPK, ERK, PTEN, PI3K, AKT, this cluster has been associated with mechanisms leading to coronary heart disease, myocardial infarction, and cardiac aging (99).

Concerning circulating miRNAs deregulated in APS patients, miR-133 and miR-145 have also shown high potential as biomarkers for both, diagnosis and prognosis for survival in patients with coronary artery disease, atherosclerosis, and acute coronary syndrome. They regulate targets related to angiogenesis, endothelial function, apoptosis and differentiation of both vascular smooth muscle cells and cardiac myocytes (100).

Altogether, miRNAs have a versatile range of abilities to manipulate posttranscriptional mechanisms leading to regulate coding genes engaged in different but interrelated diseases, including autoimmune and cardiovascular disorders, thus showing that they could play a regulatory role in the common pathways shared by these diseases. Therefore, the analysis of the miRNA expression profile could be a useful tool to identify biomarkers able to early recognize patients prone to develop more severe complications. At the same time, the identification of specific altered miRNAs might allow the identification of specific pathogenic pathways and suggest new treatment strategies.

## Novel Therapeutic Options for Managing Thrombosis in Patients With Antiphospholipid Syndrome

As more insight is gained about the pathophysiology of APS and the involvement of receptors, intracellular pathways and genetic and epigenetic alterations, new treatment modalities have been proposed, on which the patient's cellular and molecular profiles are becoming more and more relevant.

## Pharmacogenomic Studies Developed in APS Patients With CV Risk

In autoimmune diseases, such as APS, pharma genomics has led to several DNA-based tests to improve drug selection, adjust dosing, and diminish the risk of toxicity.

It has been shown that the cytochrome P450 (CYP) 2CP inactivates warfarin -the most common oral anticoagulant drug for APS-, but there is a percentage of population carrying variants in the CYP2CP gene that confer low enzyme activity (called ^*^2 or ^*^3 variants). These patients require significantly lower doses of warfarin, and even have risk of life-threatening bleeding when take standard doses (101). In this regard, a study by Kondrat'eva et al. (102) evaluated in 88 APS patients the estimated frequency of thrombotic and hemorrhagic complications during moderately intensive therapy with warfarin. They found that mutant cytochrome P450 gene variants (CYP2C9^*^2 and 3) were present in 38.5% of the patients. The number of nasal and gingival hemorrhages was increased in patients with CYP2C9^*^3. That study concluded that the determination of CYP2C9 genotype in APS patients before warfarin use might allow to avoid excessive hypo coagulation and related hemorrhages.

On the other hand, a number of genes have been reported to exhibit polymorphisms that influence the response to antithrombotic/anti-inflammatory drugs. For example, common single nucleotide polymorphisms (SNPs) in the 3-hydroxy-3-methylglutaryl-CoA reductase (HMGCR, statins) gene (a A-T substitution at position 74726928 and a T-G substitution at position 74739571) were related to differential response to pravastatin treatment (103), so that individuals with a single copy of the minor allele of these SNPs showed reduced to 22% the overall efficacy for modifying total cholesterol concentration.

Other pleiotropic genes whose changes have been demonstrated with statins are the angiotensin-converting enzyme (ACE) gene, the b-fibrinogen (FGB) gene, the glycoprotein IIIa (Gilia) gene, the stromelysin-1 (MMP3) gene, the CD36 gene, and the estrogen receptor a (ESR1) gene (104).

That overall data suggest that genetic diagnostic tests might help to identify patients at high risk for adverse drug reactions, allowing to personalized dosage modifications and thus making medications safer.

## Genes and miRNAs as Potential Biomarkers for Thrombosis Monitoring in APS

A recent study (5) demonstrated that aPLs can disrupt the mitochondrial function of monocytes and neutrophils, leading to the generation of various intracellular ROS and the subsequent expression of TF and other proinflammatory cytokines. The inhibition of intracellular ROS in monocytes with the use of Coenzyme Q10 (CoQ10, an antioxidant with anti-inflammatory and anti-thrombotic properties), *in vitro*, prevented the upregulation of TF and VEGF/Flt-1 induced by IgG-aPL.

Previous studies have proven that changes in mitochondrial morphology and function may affect several features of cardiovascular biology. Moreover, inhibiting mitochondrial fission has been reported to be cardioprotective (105). Accordingly, we showed that CoQ10, *in vitro*, prevented mitochondrial dysfunction (involving both fission and altered mitochondrial membrane potential), oxidative stress, and the suppressed the expression of prothrombotic markers relevant to the pathophysiology of APS.

Subsequently, we analyzed the potential *in vivo* beneficial effects of CoQ_10_ supplementation in the prevention of athero-thrombosis in APS patients, by developing a clinical trial (Clinical Trials.gov: NCT02218476). To develop this study (91), thirty-six APS patients were randomized to receive the reduced form of coenzyme Q10 (ubiquinol or Qred; 200 mg/d) or placebo for 1 month. The results showed that Qred modulated the overexpression of prothrombotic and proinflammatory mediators along with the improvement on endothelial function, and the reversion in the expression and/or activity of thrombosis-related protein kinases, peroxides levels, mitochondrial function, and NETosis process.

In parallel, the effect of Q_red_ treatment in the miRNA profile of monocyte from APS patients was evaluated, along with their relationship with the changes observed on the inflammatory and prothrombotic profile. By using Nano string array technology and RT-PCR validation analysis, we identified for the first time in APS, the altered mRNA and miRNA signatures of monocytes related to atherosclerosis. Monocytes gene profiling showed differential expression of 29 atherosclerosis-related genes. In parallel, 21 miRNAs were found differentially expressed in relation to healthy donors, including 18 reduced and 3 increased, whose functional classification indicated preponderance in processes such as inflammatory response, reproductive system disease, and connective tissue disorders. Q_red_ treatment for 1 month promoted a significant reversion of the expression levels of 23 mRNAs and 16 miRNAs. The interaction network miRNA- mRNA demonstrated that the presence of several Q_red_-upregulated microRNAs seemed to control simultaneously the expression of various Q_red_-downregulated genes. Thus, we have identified novel and specific miRNA–mRNA regulatory networks, related to CVD in patients with APS and modified by effect of Qred. Altogether, these results might open the scenario for the search of specific miRNAs as novel biomarkers of response to treatment and monitor of disease in APS.

## Conclusions

In recent years, there have been many advances in the understanding of the molecular basis for vascular involvement in APS, but many areas need to be further investigated, in particular the association between altered genetic/epigenetic profiles, autoantibodies and clinical manifestations, and the effectiveness of new therapeutic strategies.

It would be interesting to apply next generation sequencing technologies like RNA-Seq along with GWAS to screen both, the gene profile and the whole transcriptome of large cohorts of primary APS patients, in order to reveal the mutations/polymorphisms, post-transcriptional modifications, and changes in the gene expression as compared to healthy controls, and their relationship with the risk of thrombosis. Additionally, epigenomic studies (DNA methylation, histones modifications and miRNA profiles) on patients with primary APS would help to identify and better characterize the regulatory mechanisms that influence the abnormal expression and activities of the genes contributing to inflammation, thrombosis and organ damage in primary APS.

To date, a vast number of genetic and epigenetic biomarkers have been identified and probed to be specifically associated to the main clinical features of APS patients. Although no study has delineated which biomarkers could be considered as the most clearly associated with the highest risk of thrombosis, emerging studies are evaluating, by using bioinformatic analyses and based on a significant number of previous works, the genetic risk factors that most significantly contribute to the development of thrombosis in primary APS. As described in a precedent section, a recent study allowed to find 16 genes as the most clearly involved, including a number of them that mostly regulate the immune system and the blood coagulation pathways (CXCL4L1, P-Selectin, TLR2, TLR4, PAI-1, β2GPI, GP1a, GP1BA, PAR1, PAR2, TFPI, TF, VEGFA, FLT1, TNF, and prothrombin). In parallel, several studies have defined a number of miRNAs (mir-19b, miR20a, miR-124a-3p, miR-125a-5p, miR-125b-5p, miR-146a-5p, miR-155- 5p, and miR-222-3p), altered in their expression in key cells involved in the development of thrombosis (i.e., monocytes and neutrophils) or found deregulated in the plasma of thrombotic APS patients (miRNAs 34a-5p, 15a-5p, 133b-3p, 145a-5p, 124-3p, 20a-5p, 19b-3p, 210-3p, 206, 296-5p, and 374a-5p). Interestingly, those miRNAs were demonstrated to regulate the expression of the above defined as “*the highest prothrombotic genes*.” Furthermore, all of them were deregulated by effect of antiphospholipid antibodies, of which it has been shown that their persistent and redundant presence (i.e., triple positivity) along with their titres, directly influence the highest risk of thrombosis.

Thus, although further wider studies are required to definitively demonstrate that coordinated deregulation in APS patients, and even when other molecular actors such as polymorphisms might influence these alterations, these achievements in our understanding of the disease have opened the door to the possibility of new model targeted therapeutic options for the prevention of thrombotic events in APS.

## Author Contributions

CL-P and CP-S provided initial planning and wrote sections of the manuscript, edited the text, and gave final approval. NB, MA, AP-T, and EC participated in the planning and writing of sections of the manuscript, edited the text, and gave final approval.

### Conflict of Interest Statement

The authors declare that the research was conducted in the absence of any commercial or financial relationships that could be construed as a potential conflict of interest.
